# Efficacy and safety of traditional Chinese herbal formula combined with western medicine for uterine fibroid: A protocol for systematic review and meta-analysis

**DOI:** 10.1097/MD.0000000000022039

**Published:** 2020-09-04

**Authors:** Yu Fu, Yihua Fan, Wei Fan, Yubing Lv, Siyu Ai, Chenghao Yu

**Affiliations:** a Chengdu University of Traditional Chinese Medicine, Chengdu, Sichuan province; b Ethnic Medicine Research Institute of Southeast of Guizhou province, Kaili, Guizhou province; c First Teaching Hospital of Tianjin University of Traditional Chinese Medicine; d Tianjin University of Traditional Chinese Medicine, Tianjin, China.

**Keywords:** Chinese herbal formula, meta-analysis, protocol, systematic review, uterine fibroid

## Abstract

**Background:**

Clinical studies found that the combination of traditional Chinese herbal formula, and western medicine therapy are better in shrinking fibroids and improve other symptoms. This study aims to systematically evaluate the efficacy and safety traditional Chinese herbal formula combined with western medicine in the treatment of uterine fibroids.

**Methods:**

Randomized controlled trials of traditional Chinese herbal formula combined with western medicine for uterine fibroids patients will be searched in PubMed, Medline, Embase, Cochrane Library, China National Knowledge Infrastructure (CNKI), Chongqing VIP Chinese Science and Technology Periodical Database, Chinese Biological and Medical database (CMB), and Wanfang database from inception to August 2020. Two researchers will perform data extraction and risk of bias assessment independently. Statistical analysis will be conducted in RevMan 5.3.

**Results:**

This study will summarize the present evidence by exploring the efficacy and safety of traditional Chinese herbal formula combined with western medicine in the treatment of uterine fibroids

**Conclusions:**

The findings of the study will help to determine potential benefits of traditional Chinese herbal formula combined with western medicine in the treatment of uterine fibroids.

**Ethics and dissemination:**

The private information from individuals will not be published. This systematic review also will not involve endangering participant rights. Ethical approval is not required. The results may be published in a peer-reviewed journal or disseminated in relevant conferences.

**OSF Registration number:**

DOI 10.17605/OSF.IO/XUA8V.

## 1 Introduction

Uterine fibroids, also known as uterine leiomyomas or fibroids, are the most common benign tumors of the uterus. They are more common in women at the stage of childbearing age, with an incidence rate of 20% to 25%. Most women have no symptoms while others might suffer abnormal uterine bleeding, pelvic compression, pain, or even infertility, and surgery or medication is generally used for treating fibroids.^[1–3]^ For women who want to retain their uterus, myomectomy is usually recommended when conservative treatment fails. While there is still a high recurrence rate after myomectomy.^[4,5]^ Hysterectomy is recommended only as the last option, which has obvious therapeutic effects without recurrence. However, it could lead to ovarian insufficiency, which will easily accelerate senility and osteoporosis, or causing depression.^[6,7]^

Many western medications could be used to control symptoms of uterine fibroids, such as non-steroidal anti-inflammatory drugs (NSAIDs), gonadotropin-releasing hormone analogs, mifepristone, progesterone, and ulipristal acetate.^[8–10]^ The short-term effect of those medications is definite, but they are limited by unpleasant side effects, such as estrogen deficiency, transient postmenopausal symptoms. Also, in many cases, the fibroids will regrow after cessation of the medications.^[11–13]^ In order to alleviate the side effects of western medications, patients with uterine fibroids often turn to traditional Chinese medicine or a combination of traditional Chinese herbs and western medicine in China.^[14,15]^

In China, it has been a long history of treating uterine fibroids with Traditional Chinese medicine, which is also a widely used alternative therapy. Modern pharmacological researches showed that a variety of Chinese herbal medicines against uterine fibroids could function in reducing blood viscosity, anti-platelet aggregation, enhancing immunity, regulating endocrine, anti-inflammatory, analgesic, and anti-tumor effect.^[16,17]^ Clinical studies also found that the traditional Chinese herbal formula could not only shrink fibroids, but also it could improve the symptoms of patients with irregular bleeding, pain, irregular menstruation, and reduce the side effects of western medications.^[15,17–19]^ In recent years, there are many systematic reviews focusing on evaluating the efficacy of just Chinese herbs or western medicines,^[14,20]^ but with few meta-analysis of Chinese herbal formula combined with western medicine. Therefore, this study aims to systematically evaluate the efficacy and safety traditional Chinese herbal formula combined with western medicine in the treatment of uterine fibroids.

## 2 Methods

### 2.1 Study registration

This protocol of systematic review and meta-analysis has been drafted under the guidance of the preferred reporting items for systematic reviews and meta-analyses protocols (PRISMA-P).^[22]^ Moreover, it has been registered on open science framework (OSF) on July 31, 2020 (Registration number: DOI10.17605/OSF.IO/XUA8V).

### 2.2 Ethics

Ethical approval is not required because there is no patient recruitment and personal information collection, and the data included in our study are derived from published literature.

### 2.3 Inclusion criteria for study selection

#### 2.3.1 Type of studies

Randomized controlled trials (RCTs) of Chinese herbal formula combined with western medicine the treatment of uterine fibroids will be included. The language will be limited to Chinese and English.

#### 2.3.2 Type of participants

All the included cases conform to the diagnosis of uterine fibroids,^[21]^ regardless of nationality, race, age, gender, and source of cases.

#### 2.3.3 Type of interventions

The study focuses on RCTs of uterine fibroids treated with Chinese herbal formula combined with western medicine vs western medicine, and the type of western medicine, dosage, and the duration of treatment will not be limited.

#### 2.3.4 Type of outcome measures

Primary outcomes are relief of symptoms: abnormal uterine bleeding, pain, and pressure measured by patient self-reports or scales. Secondary outcomes includes reduction in fibroid size and uterine volume measured by ultrasonography or Magnetic Resonance Imaging (MRI), live birth, Uterine Fibroid Symptom and Quality of Life (UFS-QOL) assessment: symptom severity, control of heavy menstrual blood loss, pregnancy rate, occurrence of adverse events, and cost-effectiveness.

### 2.4 Exclusion criteria

1. Studies with unsatisfactory outcome indicators, or the outcomes of interest were not clearly reported;
2. As for duplicate published literatures, select the literature with the most complete data;
3. Extracting the related data from the published results is impossible, and unable to obtain the literature after contacting the author;
4. Literature with errors in random methods.

### 2.5 Search strategy

The PubMed, Medline, Embase, Cochrane Library, China National Knowledge Infrastructure (CNKI), Chongqing VIP Chinese Science and Technology Periodical Database, Chinese Biological and Medical database (CMB), and Wanfang database will be retrieved for all relevant randomized controlled trials (RCTs) until August 2020. The reference lists of relevant articles will be reviewed for eligible inclusion.

The search terms and combination of keywords are “Chinese herbal formula”, “traditional Chinese medicine”, “uterine fibroids”, “uterine leiomyomas”, etc. The search terms will be revised according to the rule of each database. The search strategy of PubMed is listed in Table 1.

**Table 1:**
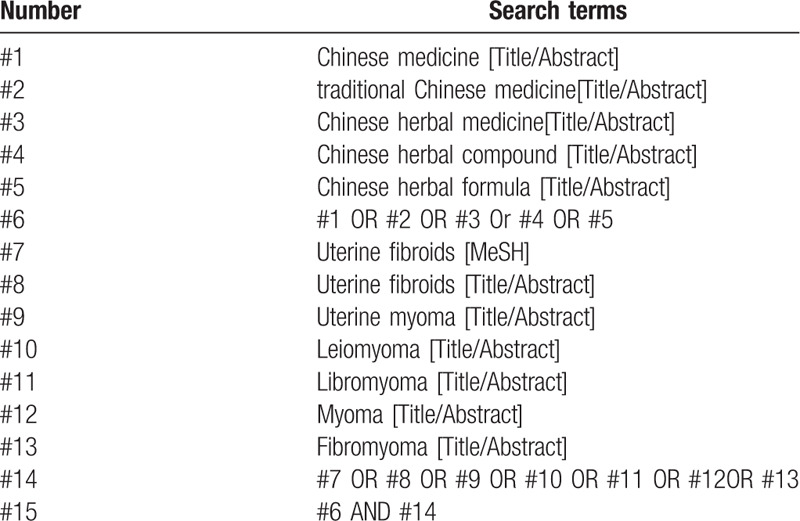
Search strategy in PubMed database.

### 2.6 Data extraction

**Figure 1:**
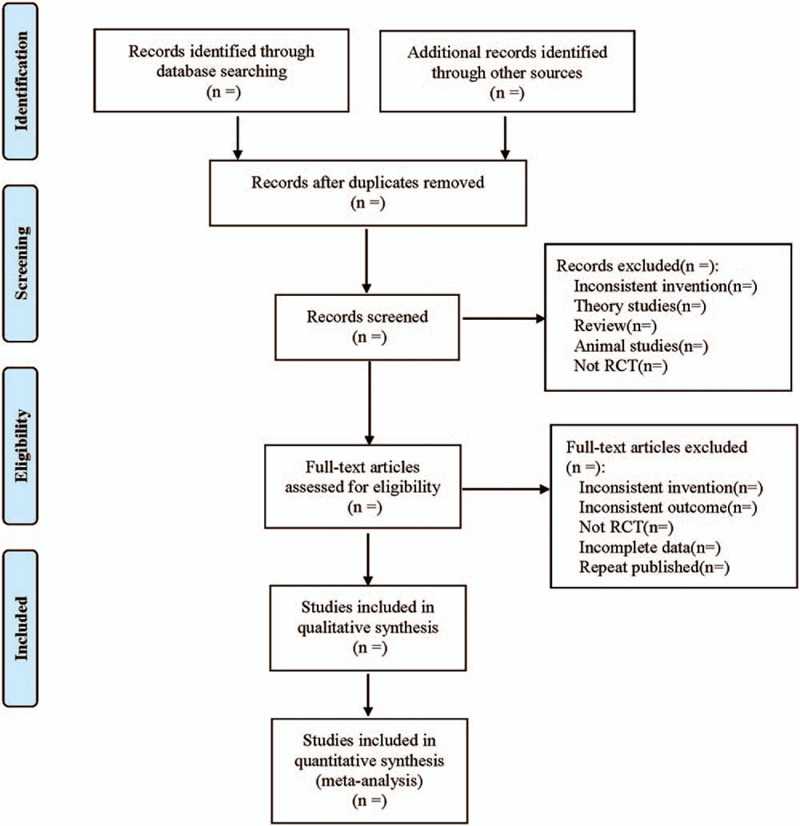
Flow diagram.

All the literature meeting the criteria will be obtained and managed by Endnote X^7^. The literature screening process is shown in Figure 1. Two reviewers will separately complete the extraction. Under the instruction of the Cochrane Collaboration, Excel 2019 is used to set up a data extraction table to extract data, including study identification (title, authors, journal, publication year, and country), participants information (age, sample size, sex ratio, course of disease), randomization method, concealment, interventions in treatment, and control groups, outcomes, adverse events, and other details. Discrepancies will be resolved through discussion or consulting a third reviewer.

### 2.7 Risk of bias assessment

Two researchers independently will evaluate the risk of bias in randomized controlled trials in accordance with the Cochrane Handbook of Systematic Reviewers, including the following items: random sequence generation, allocation concealment, blinding of participants and personnel, blinding of outcome assessment, incomplete outcome data, selective reporting, and other bias. The quality of studies is classified as being at of high, unclear or low risk of bias. In case of disagreement, a third researcher will decide.

### 2.8 Statistical analysis

#### 2.8.1 Data synthesis

The RevMan 5.3 software provided by the Cochrane Collaboration will be used for statistical analysis. ① Relative risk (RR) is selected as the statistic for the dichotomous variable. For continuous variables, weighted mean difference (WMD) is selected when the tools and units of measurement indicators are the same, standardized mean difference (SMD) is selected with different tools or units of measurement, and all the above are represented by effect value and 95% Confidence interval (CI). ② Heterogeneity test: Q test is used to qualitatively determine inter-study heterogeneity. If *P* ≥ .1, there is no inter-study heterogeneity; If *P* < .1, it indicate inter-study heterogeneity. At the same time, *I*^*2*^ value is used to quantitatively evaluate the inter-study heterogeneity. If *I*^*2*^ ≤ 50%, the heterogeneity is considered to be good, and the fixed-effect model is adopted. If *I*^*2*^ > 50%, it is considered to be significant heterogeneity, the source of heterogeneity will be explored through subgroup analysis or sensitivity analysis. If there is no obvious clinical or methodological heterogeneity, it will be considered as statistical heterogeneity, and the random-effect model will be used for analysis. Descriptive analysis will be used if there is significant clinical heterogeneity between the 2 groups and subgroup analysis is not available.

#### 2.8.2 Dealing with missing data

If data is missing or incomplete in a study, the corresponding author will be contacted to obtain the missing data. If impossible, the study will be removed.

#### 2.8.3 Heterogeneity and subgroup analysis

In order to reduce the clinical heterogeneity between studies, subgroup analysis is conducted according to patient age or size of fibroid. Moreover, subgroup analysis could also be carried out according to the different western medicines in the treatment group.

#### 2.8.4 Sensitivity analysis

In order to test the stability of meta-analysis results of indicators, a one-by-one elimination method will be adopted for sensitivity analysis.

#### 2.8.5 Reporting bias

For the major outcome indicators, if the included study is ≥10, funnel plot will be used to qualitatively detect publication bias. Eggers and Beggs test are used to quantitatively assess potential publication bias.

#### 2.8.6 Evidence quality evaluation

The Grading of Recommendations Assessment, Development, and Evaluation (GRADE)^[23]^ will be used to assess the quality of evidence. It contains 5 domains (bias risk, consistency, directness, precision, and publication bias). And the quality of evidence will be rated as high, moderate, low, and very low.

## 3 Discussion

The mechanism of uterine fibroids is still not clear, and it is known that genetics, hormones, immune system, and environment play an important role in the growth of uterine fibroids. In the traditional Chinese medicine theory, uterine fibroids are related to the imbalance of qi and blood. The commonly used herbs are *radix paeoniae rubra(Chi Shao)*, *panax notoginseng(San Qi*), *peach kernel(Tao Ren)*, *cassia twig(Gui Zhi)*, *poria cocos(Fu Ling)*, etc. Modern pharmacological studies have shown that *radix paeoniae rubra* could inhibit tumor cell membrane activity, improve blood microcirculation, increase adenosine, and cyclase, play an anti-tumor effect;^[23,24]^*panax notoginseng* has the effects of inhibiting tumor cell growth, and inducing tumor cell apoptosis.^[25,26]^ The combination of multiple herbs formula could greatly improve the efficacy and safety to treat uterine fibroids when combined with western medications to influence the hormone environment and inhibit the growth of tumors, such as leuprolide, mifepristone, and triptorelin. Clinical studies have also found that the combination of traditional Chinese herbal formula and western medicine therapy are better in shrinking fibroids and improve other symptoms such as bleeding irregularities, subfertility, and preterm birth.^[27]^

Yet, the efficacy and safety of Chinese herbal formula combined with western medicine in treating uterine fibroids still needs evidence of systematic review and meta-analysis. We try to conduct this systematic review tend to figure out the efficacy and safety of the combined therapy. This meta-analysis and systematic review will help to determine potential benefits of Chinese herbal formula combined with western medicine compared with different western medicines to treat women with uterine fibroids. However, the study has some limitations. Our search did not include studied in other languages except Chinese and English, which might result in certain selective bias. In addition, due to the different herbal formulas and western medicines used in the treatment of different studies, there might be some heterogeneity.

## Author contributions

**Data collection**: Yu Fu and Yihua Fan.

**Funding support**: Chenghao Yu.

**Literature retrieval**: Wei Fan and Yubing Lv.

**Software operating**: Siyu Ai.

**Supervision**: Chenghao Yu.

**Writing** – **original draft**: Yu Fu and Yihua Fan.

**Writing** – **review & editing**: Yu Fu and Chenghao Yu.
